# Role of Tranexamic Acid in Controlling Blood Loss in Hemiarthroplasty of the Hip: A Prospective and Observational Study

**DOI:** 10.7759/cureus.62617

**Published:** 2024-06-18

**Authors:** Hamdi Nizar Ahamed, Sandeep Mohan, Rahul Krishnan

**Affiliations:** 1 Orthopedics and Traumatology, Government Medical College, Kannur, IND; 2 Orthopedics and Traumatology, St. Joseph's Hospital Karuvanchal, Kannur, IND

**Keywords:** prospective study, hemoglobin drop, blood loss, hemiarthroplasty of hip, tranexamic acid

## Abstract

Introduction: Tranexamic acid, an antifibrinolytic drug, is well-established for its efficacy in reducing intraoperative and postoperative blood loss in major orthopedic surgeries, particularly total knee replacement (TKR) and spine surgeries. However, there is limited research on the role of tranexamic acid in hemiarthroplasty of the hip. This study aims to investigate the efficacy of tranexamic acid in controlling blood loss in hemiarthroplasty of the hip.

Objectives: The primary objective was to analyze the pre- and postoperative changes in hemoglobin levels among patients undergoing hemiarthroplasty of the hip with and without intravenous tranexamic acid administration.

Methods: A prospective observational study was conducted at the Department of Orthopedics of Government Medical College, Kannur, and St. Joseph’s Hospital, Karuvanchal, Kannur. Patients undergoing hemiarthroplasty of the hip with 1 gm of intravenous tranexamic acid were compared with those without tranexamic acid. The sample size was calculated to be 33 in each group. Data were collected using a standardized proforma, entered into an Excel sheet (Redmond, USA), and analyzed using IBM Corp. Released 2017. IBM SPSS Statistics for Windows, Version 25.0. Armonk, NY: IBM Corp.

Results: The study comprised mainly elderly females with a mean age of 74.84 ± 8 years. There was a significant reduction in postoperative hemoglobin drop, requirement for postoperative blood transfusion, and total drain output for the initial three postoperative days among patients who received intravenous tranexamic acid.

Conclusion: Preoperative administration of intravenous tranexamic acid significantly reduced postoperative hemoglobin drop and the need for postoperative blood transfusion in patients undergoing hemiarthroplasty of the hip. This highlights the efficacy and safety of tranexamic acid in controlling blood loss in this surgical setting.

## Introduction

Orthopedic surgeries, particularly joint arthroplasty, have long been associated with substantial blood loss, posing significant risks of morbidity and mortality [1]. Consequently, a considerable number of patients require blood transfusions post-surgery, which further heightens the risk of complications [2]. While blood transfusion is crucial in cases of hemorrhage, it is costly and often faces challenges such as scarcity of sources and the potential for infections and immune reactions, despite screening measures [2]. Moreover, factors like availability, cost, shelf life, and religious concerns add to the obstacles to utilizing blood transfusions [2]. In orthopedic surgeries, effective management of blood loss is paramount and has evolved alongside surgical techniques [3]. Achieving adequate intraoperative hemostasis, especially in surgeries involving the hip joint, is essential to prevent complications like hematoma formation and excessive blood loss via suction drain. Furthermore, attaining satisfactory postoperative hip joint mobility hinges on the hemostasis achieved during surgery. Therefore, reducing operative blood loss not only enhances patient outcomes but also curtails healthcare costs [4]. Various strategies for blood conservation have been employed, including autologous blood donation, recombinant erythropoietin, platelet-rich plasmapheresis, acute normovolemic hemodilution (ANH), cell salvage (scavenging), fibrin sealants, and anti-fibrinolytic [3]. Among these strategies, tranexamic acid (TA) stands out as a revolutionary antifibrinolytic agent extensively utilized during surgeries to control intraoperative blood loss, such as in coronary artery bypass surgery and dental procedures. Compared to alternatives like erythropoietin (EPO) or iron supplementation, TA has proven to be both less expensive and more effective in blood management [3]. Studies have shown that TA is not only cost-effective but also superior to reinfusion drains and PAD (perioperative autologous donation) [5], thus holding immense potential to revolutionize blood management in orthopedic surgeries [6]. Tranexamic acid, a synthetic antifibrinolytic, exhibits rapid absorption in the body, with approximately 90% excretion through urine within 24 hours. It has a plasma half-life of about 2-3 hours, maintaining therapeutic levels for 6-8 hours [7,8]. Both topical and intravenous administrations of TA have been documented in the literature, consistently demonstrating reductions in perioperative blood loss and transfusion requirements. While previous studies have extensively investigated the efficacy of TA in reducing blood loss and transfusion requirements in surgeries like total knee replacement, total hip replacement, and spine surgeries [9-11], research on its application in hemiarthroplasty of the hip remains limited. Hemiarthroplasty of the hip is a commonly performed surgery for displaced femoral neck fractures, particularly in elderly patients with lower functional demands [11]. Given the heightened perioperative risks associated with elderly patients due to comorbidities, an investigation into the role of TA in hemiarthroplasty of the hip is warranted. However, compared to knee arthroplasty and total hip replacement, studies on the use and effects of TA in hemiarthroplasty of the hip are sparse. Therefore, this study aims to address this gap and evaluate the role of TA in reducing blood loss in hemiarthroplasty of the hip.

## Materials and methods

This prospective observational study was conducted on patients with fractures of the neck and femur admitted to the Department of Orthopedics at Government Medical College Kannur and St. Joseph’s Hospital Karuvanchal after getting written informed consent.

Study period: November 2019 to November 2020.

From the previous study [10], the sample size was calculated using the nMaster software by following the following formula:

n = (Zα+Zβ)2/∆2 +Zα/2,

effect size = μ2-μ1/SD, SD=(SD1+SD2)/2

μ1=pre-test mean, μ2=post-test mean

Zα=1.96 Zβ = 0.84

SD: 1.3 d:0.5

Sample size-33 in each group

Sampling technique-consecutive sampling

Inclusion and exclusion criteria are listed in Table 1.

**Table 1:** Inclusion and exclusion criteria

INCLUSION CRITERIA	EXCLUSION CRITERIA
All cases who underwent hemiarthroplasty of hip	Patients with a history of allergy to tranexamic acid
	Patients with pre-existing bleeding disorder
	Revision surgery
	Acute infections

Control group: data was collected through case sheets of patients from the medical records department who had undergone hemiarthroplasty of the hip (via the Hardinge approach) without the infusion of tranexamic acid.

Study tools: pre- and postoperative hemoglobin difference, number of units of blood transfusions done postoperatively, drain output (for the initial three postoperative days), and number of mops used during surgery.

Study procedure: Ethics committee approval was obtained from the institutional ethics committee. Subsequently, consecutive patients scheduled to undergo hemiarthroplasty of the hip were included in the study, adhering to the predefined inclusion and exclusion criteria. Written informed consent was obtained from all participants before their inclusion in the study. Pertinent details, including preoperative hemoglobin levels, were meticulously recorded. Patients undergoing hemiarthroplasty of the hip via the Hardinge approach and receiving preoperative intravenous administration of 1gm of tranexamic acid (administered half-hourly before incision) were assessed using predetermined measurement tools. The data obtained from these participants were then compared with those of the non-tranexamic acid group, consisting of patients who did not receive preoperative intravenous tranexamic acid. Subsequently, a comprehensive statistical analysis was conducted on the collected data. This analysis involved the coding and entry of data into Microsoft Excel, followed by analysis using IBM Corp. Released 2017. IBM SPSS Statistics for Windows, Version 25.0. Armonk, NY: IBM Corp. Categorical variables such as age category, gender, addictions, and comorbidities were expressed as percentages, while continuous variables such as age and surgery time were presented as mean and standard deviation. For quantitative data with a normal distribution, the Student's t-test was utilized, whereas the Mann-Whitney U test was employed for data lacking a normal distribution. The significance of categorical variables was assessed using the chi-square test. The strength of the association was quantified using odds ratios with corresponding confidence intervals. A p-value of less than 0.05 was deemed statistically significant.

## Results

Thirty-three patients who underwent hemiarthroplasty of the hip were administered intravenous tranexamic acid preoperatively (half an hour before surgery). They were compared with 33 patients who didn’t receive tranexamic acid before hemiarthroplasty of the hip. A group of patients who underwent hemiarthroplasty of the hip and were administered intravenous tranexamic acid preoperatively (half an hour before surgery) will henceforth be referred to as the Tranexamic Acid Group (TA Group). Patients who didn’t receive tranexamic acid before hemiarthroplasty of the hip will henceforth be referred to as the non-tranexamic acid group (non-TA group).

The mean age of the study participants is 74.84 ± 8 years. Thirteen (39.3%) of the participants in the TA group were males, and 20 (60.7%) were females, compared to 15 (45.5%) males and 18 (54.5%) females in the non-TA group. The addiction status of the participants was as follows: 26 (78.8%) participants in the TA group had no addictions, 5 (15.2%) had a smoking addiction, one (3%) had an alcohol addiction, and one (3%) had both addictions. In the non-TA group, 25 (76%) participants had no addictions, two (6%) were addicted to smoking, three (9%) to alcohol, and three (9%) participants were addicted to both. Among the TA group, 60% of patients (20 patients) had comorbidities, while among the non-TA group, 75.8% (25 patients) had comorbidities. The mean surgery time among patients who received IV tranexamic acid was 101.06 ± 11.16 minutes, while for patients who didn’t receive IV tranexamic acid, the mean surgery time was 104.55 ± 9.38 minutes. There is no significant difference in the mean surgery time between those who received IV tranexamic acid (101.06, 11.16) and those who did not (104.55, 9.38); t(64) = -1.37, p=0.175. There is a significant difference in the median number of mops used during surgery between those who received IV tranexamic acid (3, IQR, 3-4) and those who did not (4-4.5); U=943.5, p=<0.001. The requirement for blood transfusions post-surgery was as given in Table 2. 

**Table 2:** Requirement of blood transfusion post-surgery

	BLOOD TRANSFUSION REQUIRED (NUMBER OF PATIENTS)		TOTAL (NUMBER OF PATIENTS)
	YES	NO	
TA group	7	26	33
Non-TA group	18	15	33
TOTAL	22	44	66

A chi-square test of independence was performed to examine the relationship between post-surgery blood transfusion requirements and tranexamic acid administration. The relation between these variables was significant: X2 (1,) = 7.79, p = 0.

The number of units of blood transfused post-surgery is given in Table 3. 

**Table 3:** Number of units of blood transfused post-surgery

NO OF UNITS OF BLOOD TRANSFUSED POST-SURGERY	TA GROUP (NUMBER OF PATIENTS)	NON-TA GROUP (NUMBER OF PATIENTS)
0	26	15
1	7	15
2	0	3

**Figure 1:**
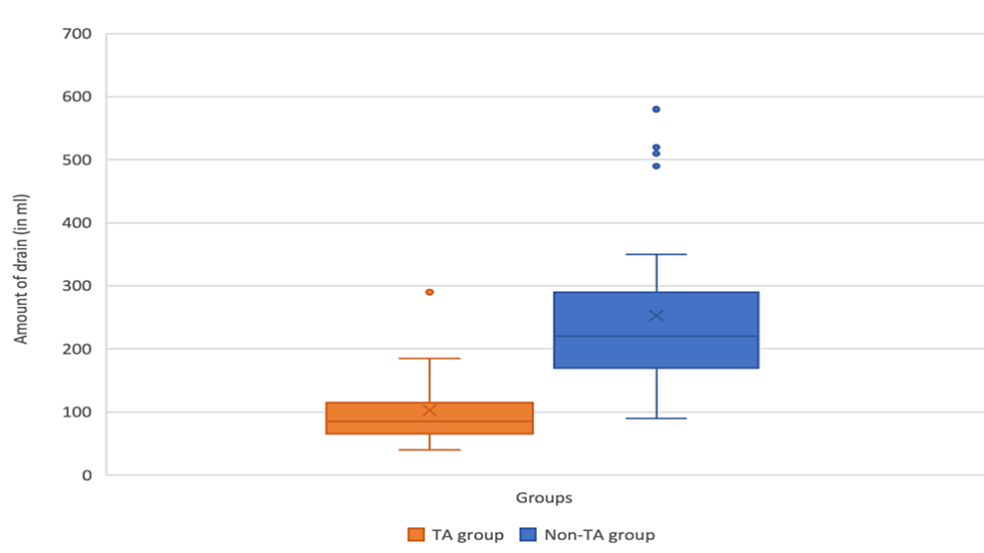
Box whisker plot depicting the total drain amount for the initial three consecutive postoperative days

The total drain amount for the first three postoperative days was as depicted in the box whisker plot (Figure 1).

The median total drain for the initial three postoperative days among the TA group was found to be 85 ml (IQR,65-115) and 220 ml (IQR,165-292.5) among the non-TA group. This difference was found to be statistically significant on the Mann-Whitney U test (U=989.5; p<.001).

The mean preoperative hemoglobin value in the TA group is 12.02 ±1.02 g/dl, and in the non-TA group, it is 11.84 ± 1.12 g/dl. The mean hemoglobin value for the initial three postoperative days in the TA group is 11.17 ± 1.05 g/dl, and in the non-TA group, it is 10.26 ± 1.21 g/dl (Table 4).

**Table 4:** The preoperative and postoperative mean hemoglobin values

Mean Haemoglobin values	TA GROUP	NON-TA GROUP
Preoperative(g/dl)	12.02 ± 1.02	11.84 ± 1.12
Postoperative day1(g/dl)	11.55 ± 0.84	10.51± 1.31
Postoperative day2(g/dl)	11.15 ± 0.83	10.11 ± 1.39
Postoperative day3(g/dl)	10.83 ± 0.97	10.18 ± 1.31

There is an overall drop in hemoglobin values postoperatively for both those who received IV tranexamic acid and those who did not. However, the drop was less in those who received IV tranexamic acid. The median hemoglobin drop postoperatively at day one in the TA group was 0.5g/dl (IQR, 0.85-0.3), and in the non-TA group, it was 1.2g/dl (IQR, 1.65-0.55). This difference in the preoperative and postoperative hemoglobin values among the TA group and the non-TA group was found to be statistically significant (U =233, p=<.001). The median hemoglobin drop postoperatively at day two among the TA group was 0.7g/dl (IQR, 1.25-0.5), and in the non-TA group was 1.5g/dl (IQR, 2.2-0.90). This difference in the preoperative and postoperative hemoglobin values among the TA group and the non-TA group at day two was found to be significant (U =281, p=<.001). The median hemoglobin drop postoperatively at day three among the TA group was 1.1g/dl (IQR, 1.50-0.8), and in the TA group, it was 1.6g/dl (IQR, 2.45-1.00). This difference in the preoperative and postoperative hemoglobin values among the TA group and the non-TA group was found to be significant (U =389, p=<.001).

**Figure 2:**
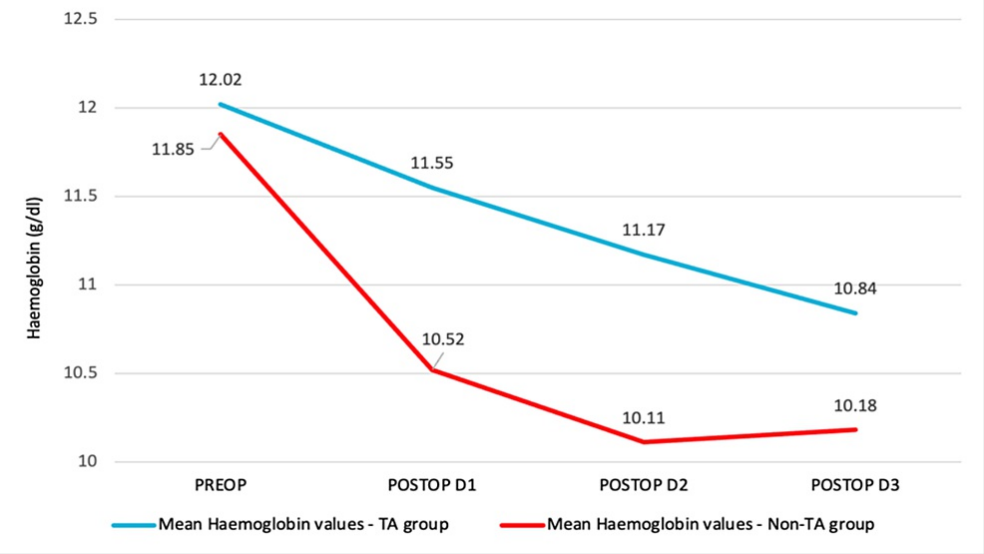
Drop in mean hemoglobin values over time

The drop in mean hemoglobin values over time is depicted in Figure 2. 

A repeated-measures ANOVA determined that mean hemoglobin values differed significantly across the time points (preoperatively, postoperatively, day 1, day 2, and day 3) between the TA group and the non-TA group (F (2.24, 143.45) = 6.14, p = .002).

The categorization of pre-postoperative hemoglobin differences in the TA and non-TA groups was as follows (Table 5).

**Table 5:** Categorization of pre-postoperative hemoglobin differences in TA and non-TA groups

	TA group, n=33	Non-TA group, n=33	P value	Odds ratio (95%CI)
PRE-POST OP Hb DIFFERENCE D1				
<1g/dl	28(84.8%)	14(42.4%)	0.000	7.60 (2.34-24.62)
>1g/dl	5(15.2%)	19(57.6%)		
PRE-POST OP Hb DIFFERENCE D2				
<1g/dl	21(63.6%)	12(36.4%)	0.027	3.06 (1.12-8.35)
>1g/dl	12(36.4%)	21(63.6%)		
PRE-POST OP Hb DIFFERENCE D3				
<1g/dl	15(45.5%)	9(27.3%)	0.125	2.22 (0.79-6.21)
>1g/dl	18(54.5%)	24(72.7%)		

A drop of >1g/dl in hemoglobin value is significantly higher among the non-TA group for postoperative days 1, 2, and 3.

**Figure 3:**
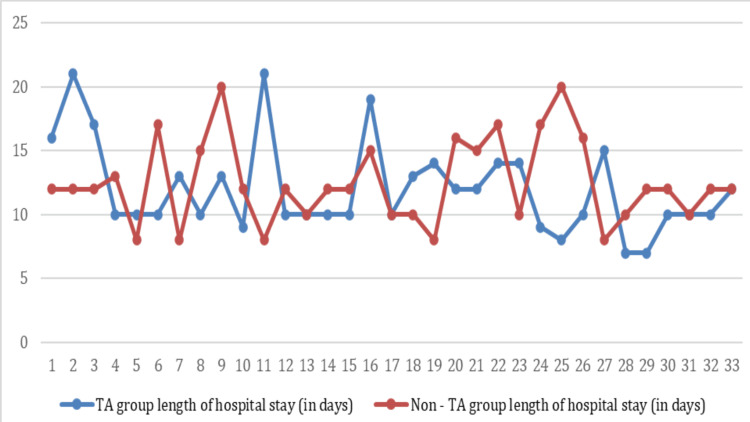
Length of hospital stay among the TA group and the non-TA group

The median duration of hospital stay among the participants who received IV tranexamic acid was 10 days (IQR, 10-14), and the median duration of hospital stay among the participants who did not receive perioperative IV tranexamic acid was 12 days (IQR, 10-15) (Figure 3).

**Figure 4:**
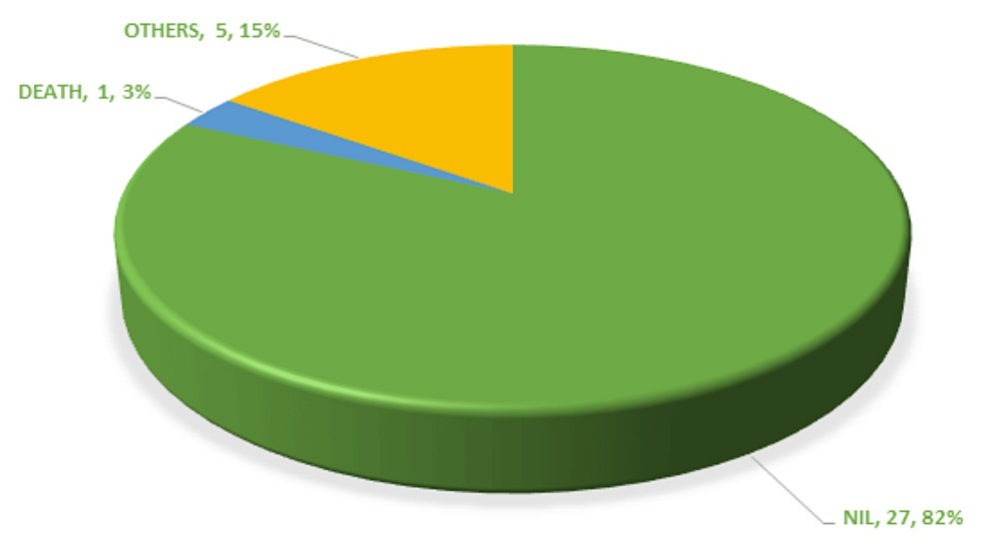
Post-operative complications among the TA group

Among those who received the perioperative tranexamic acid injection, only 18% developed postoperative complications, which included respiratory complications and one mortality (Figure 4).

**Figure 5:**
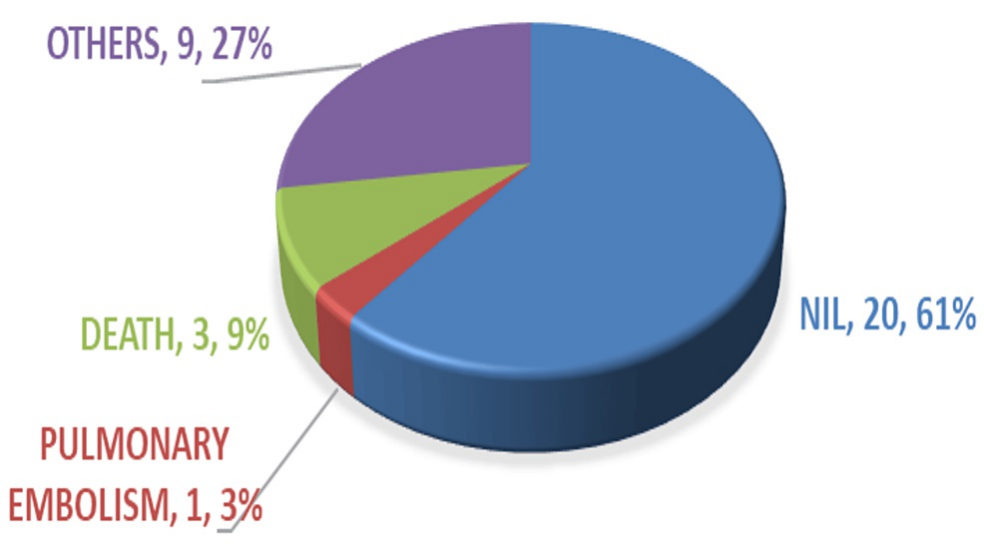
Post-operative complications among non-TA group

Among those who didn’t receive preoperative tranexamic acid injection, 39% developed postoperative complications, which included pulmonary embolism, respiratory complications, and three deaths (Figure 5).

## Discussion

Our study has demonstrated that the administration of tranexamic acid effectively mitigates blood loss in hemiarthroplasty patients across multiple parameters, including reduced need for blood transfusions, decreased units of blood transfused post-surgery, diminished drain volume, attenuated discrepancy in pre-versus post-surgery hemoglobin levels, shortened hospitalization duration, and fewer post-operative complications.

The fractured neck of the femur is a prevalent condition among the elderly, with treatment goals emphasizing early mobilization and minimal postoperative complications. Blood conservation is crucial in orthopedic surgeries due to significant intraoperative and postoperative blood loss. Tranexamic acid has emerged as a valuable tool for perioperative blood conservation, particularly in total knee replacement surgeries, but its role in other joint arthroplasties, such as hemiarthroplasty of the hip, remains less explored, especially in our country, where its use is relatively infrequent [12].

Our study aimed to address this gap by analyzing data from 33 patients who received tranexamic acid and comparing it with an equal number of patients undergoing hemiarthroplasty of the hip without the drug. Tranexamic acid was administered intravenously half an hour before surgery, aligning with evidence suggesting optimal timing for reducing postoperative blood loss [13]. The study cohort mainly comprised elderly females with a mean age of 75.5 years, consistent with previous studies [14-16]. Over 50% of patients had at least one comorbidity, and most surgeries were performed under spinal anesthesia. Notably, there was no significant difference in the mean duration of surgery between the two groups, suggesting consistency in surgical procedures. In line with previous studies, our findings demonstrated a significant reduction in postoperative blood transfusion requirements and a decrease in postoperative hemoglobin levels among patients who received perioperative tranexamic acid [17-21]. This reduction in hemoglobin levels was similar to recent research by Ashkenazi et al. [11]. However, when categorized by significant hemoglobin drop (>1 g/dl) on the third postoperative day, the difference lost significance, possibly due to postoperative allogenic blood transfusion in patients with a significant hemoglobin drop. Despite many studies reporting a reduction in postoperative complications among those in the tranexamic acid group, our study did not reveal any statistically significant difference in complication occurrence between the tranexamic acid and non-tranexamic acid groups [7]. However, the majority of patients in both groups did not experience postoperative complications, which may be attributed to standardized postoperative care. Similarly, we found no significant difference in the duration of hospital stay between the two groups, consistent with previous research [22-24]. While our study had limitations, such as not studying patients with multiple risk factors for thromboembolic events and focusing only on short-term complications during hospital stays, it achieved an adequate sample size and analyzed real-world clinical data. Thus, our study concludes that tranexamic acid effectively controls blood loss in hemiarthroplasty of the hip, adding to the existing evidence supporting its role in perioperative management.

## Conclusions

The study provides compelling evidence to support the conclusion that preoperative administration of intravenous tranexamic acid effectively controls blood loss associated with hemiarthroplasty of the hip. By significantly reducing postoperative hemoglobin drop and the need for blood transfusion, tranexamic acid not only minimizes transfusion-related complications but also contributes to cost savings in healthcare. Therefore, intravenous tranexamic acid emerges as a safe and efficient option for reducing operative blood loss and warrants consideration for routine use during hemiarthroplasty of the hip.
